# Tuberculosis Case Finding in Benin, 2000–2014 and Beyond: A Retrospective Cohort and Time Series Study

**DOI:** 10.1155/2016/3205843

**Published:** 2016-05-16

**Authors:** Serge Ade, Wilfried Békou, Mênonli Adjobimey, Omer Adjibode, Gabriel Ade, Anthony D. Harries, Séverin Anagonou

**Affiliations:** ^1^Programme National contre la Tuberculose, 01 BP 321 Cotonou, Benin; ^2^Faculté de Médecine, Université de Parakou, Parakou, Benin; ^3^International Union against Tuberculosis and Lung Disease, Paris, France; ^4^London School of Hygiene & Tropical Medicine, London, UK

## Abstract

*Objective*. To determine any changes in tuberculosis epidemiology in the last 15 years in Benin, seasonal variations, and forecasted numbers of tuberculosis cases in the next five years.* Materials and Methods*. Retrospective cohort and time series study of all tuberculosis cases notified between 2000 and 2014. The “R” software version 3.2.1 (Institute for Statistics and Mathematics Vienna Austria) and the Box-Jenkins 1976 modeling approach were used for time series analysis.* Results*. Of 246943 presumptive cases, 54303 (22%) were diagnosed with tuberculosis. Annual notified case numbers increased, with the highest reported in 2011. New pulmonary bacteriologically confirmed tuberculosis (NPBCT) represented 78%  ± SD 2%. Retreatment cases decreased from 10% to 6% and new pulmonary clinically diagnosed cases increased from 2% to 8%. NPBCT notification rates decreased in males from 2012, in young people aged 15–34 years and in Borgou-Alibori region. There was a seasonal pattern in tuberculosis cases. Over 90% of NPBCT were HIV-tested with a stable HIV prevalence of 13%. The ARIMA best fit model predicted a decrease in tuberculosis cases finding in the next five years.* Conclusion*. Tuberculosis case notifications are predicted to decrease in the next five years if current passive case finding is used. Additional strategies are needed in the country.

## 1. Introduction

Despite the discovery of the causative agent more than one century ago, a vaccine, highly effective medications, and recent improvements in biological molecular field and genetic engineering, tuberculosis (TB) remains a major public health concern in the world. In 2014, globally, there were estimated 9.6 million incident cases, of which 1.5 million were estimated to have died. The burden of the disease is particularly immense in Africa from where the case rate was reported to be 281 per 100000 people [1].

The year 2016 is an important turn for the TB world, with the World Health Organization (WHO) launching the “End TB Strategy” with ambitious new targets of reducing by 2035 the incidence and the mortality of the disease by 90% and 95%, respectively, compared with 2015 [2]. Success in this new strategy requires additional and relevant actions/strategies at both international and national levels. In countries, such actions/strategies cannot be properly planned without a clear understanding of the past and current local epidemiology of the disease.

Benin is a small country in West Africa with an area of 114763 km^2^ for a population of 10008749 inhabitants in 2013 [3]. With activities just restricted to a mass BCG vaccination before 1966, TB care activities in the country significantly improved from 1972 with the establishment of the National TB Programme (NTP). An epidemiological review of TB patients diagnosed in the country between 1995 and 2007 showed an average of notified new pulmonary bacteriologically confirmed TB (NPBCT) cases of 35 per 100000 inhabitants, with a slight increase of 1% per annum. Large variations were observed in notifications of TB cases between the north and the south of the country, in line with population densities. The male-female sex ratio was 1.8 and no change in the age structure was reported. Human Immunodeficiency Virus (HIV) prevalence of the 97% of TB patients that were tested was 14% [4].

As implementation of the “End TB Strategy” commences, it is important to better understand the new challenges and to determine whether the local TB epidemiology has changed over time. There is also no information available within the national tuberculosis control programme on the relationship between seasons and TB case finding. This information may be useful in forecasting the number of TB cases expected in Benin in the coming years.

The current study was therefore undertaken (i) to assess whether there were any changes in the local TB epidemiology the last fifteen years and (ii) to determine the forecasted number of TB cases in the next five years. Specific objectives were to determine, between 2000 and 2014, in Benin (i) the trend of presumptive and notified TB cases; (ii) the trend of the different types and categories of TB cases; (iii) any changes in baseline characteristics and HIV-status of notified NPBCT; (iv) any seasonality in TB case finding in the country; and (v) the forecasted number of TB cases that might be diagnosed within the programme in the next five years.

## 2. Materials and Methods

### 2.1. Study Design

This was a retrospective cohort and time series study of notified TB patients, using routinely collected data.

### 2.2. General Setting and Study Sites

#### 2.2.1. Country

Benin shares borders with Burkina Faso and Niger in the north, Togo in the west, Nigeria in the east, and the Atlantic Ocean in the south. The country is low-income with a Gross National Income per capita estimated at US$ 810 in 2014. The under-five and maternal mortality rates were 85 per 1000 live births and 340 per 100 000 live births, respectively, in 2013 [5]. HIV prevalence among adults aged between 15 and 49 years was 1.1% in 2012 [6]. For decades, BCG vaccination has been implemented in public and also some appointed health facilities in the country. BCG vaccination is recommended within the first two weeks after birth. In 2014, the coverage rate was 96% [7]. BCG vaccination is free of charge.

Administratively, the country is divided into six functional departments: Atacora-Donga and Borgou-Alibori in the north, Zou-Collines in the centre, and Mono-Couffo, Oueme-Plateau, and Atlantique-Littoral in the south. Cotonou the economic capital is located in Atlantique-Littoral. The density of the population gradually increases from the north to the south, with the highest population density reported from Cotonou. Overall in Benin, the climate is hot and humid. There were two rainy seasons (a principal season from April to July and a shorter season from September to November) and two dry seasons (a principal season from December to April and a shorter season from July to September).

#### 2.2.2. The National Tuberculosis Control Programme (NTP)

*Organization and Function*. The NTP is under the Ministry of Health and has a pyramidal organizational structure with three (central, intermediate, and peripheral) levels, based on the general health system model. Diagnosis, registration, and treatment are decentralized to 57 BMUs (48 public and 9 confessional health facilities) at peripheral levels. Attempts had been made previously to include private clinics but with no success, because the private sector is benefits-oriented, contrary to the programme's objective of making all TB-related activities free of charge (consultations and/or examination charges for diagnosis, treatment, and follow-up). However, these private clinics are not completely excluded from TB activities. Their workers are regularly trained to identify and refer to the closest BMU patients with presumptive symptoms of TB. This system functions well. There is supervision of all the 57 BMUs organized by the central and intermediate levels of the TB Programme on a quarterly basis.

*Diagnosis, Treatment, and Case Notification*. Diagnosis and treatment are provided in line with the WHO and The Union recommendations [8, 9]. Overall, a passive screening strategy is routinely performed, although in 2011 there was some experimentation with a semiactive case finding strategy named “TB Reach” funded by the WHO. The laboratory network is made up of 57 laboratories in BMUs with 10 additional centres for microscopic diagnosis. In these laboratories, specimens from all presumptive TB cases (regardless of their public, confessional, or private sector origin) are analysed mainly using Ziehl-Neelsen staining and/or fluorescence auramine microscopy. Fluorescent microscopy was first introduced into the largest BMU of the country twenty years ago. Since then, this technology has been scaled up to six regional laboratories in 2010 and to 10 other laboratories with the highest workloads in 2014. With the new Global Fund against AIDS, Tuberculosis and Malaria (GFATM) grant, the rest of the laboratories in the network will be equipped with fluorescent microscopy by 2017.

In addition to microscopy, phenotypic culture on solid medium (Löwenstein-Jensen) followed by identification and drug susceptibility testing is routinely carried out at the mycobacteria national laboratory. Since 2012, Xpert® MTB/RIF (Cepheid Inc., Sunnyvale, CA, USA) has also been available in this laboratory. This technology is not part of the routine diagnosis strategy of all TB cases but is mainly used for (1) detecting patients earlier with resistance to rifampicin, almost synonymous with multidrug-resistant TB and (2) diagnosing HIV-infected patients with presumptive symptoms of TB. On the first targeted population, in Benin, rifampicin resistant patients are mostly found among retreatment TB cases. Thus, all previously treated patients positive on smear microscopy are tested. In 2016, Xpert MTB/RIF (Cepheid Inc., Sunnyvale, CA, USA) was scaled up in the six regional laboratories. In the short term, decentralization of these machines at peripheral levels is not possible because of various technological and other constraints (e.g., cost, environmental temperatures, shelf-life of cartridges, electricity supplies in a country where electricity is neither stable nor permanent, and the need for annual calibration of the machine) [10].

Once the diagnosis is confirmed, patients are registered in the “TB registers” which are only available in the 57 BMUs. Those, who reside far from the place of diagnosis, are sent to the BMU closest to their domicile and registered in that new BMU.

Treatment is then started and is in line with WHO recommendations. All consultations, bacteriological examinations, treatment, and follow-up are provided free of charge. Anti-TB drugs are not available in private pharmacies but only in BMUs. This presents an advantage for TB notification completeness since patients have to be sent to one of the 57 BMUs and be registered before starting treatment. For all pulmonary TB bacteriologically confirmed cases through microscopy, treatment is directly observed during the intensive phase.

Within the first two weeks after the end of each quarter, a report on TB cases diagnosed during these three months and also outcomes of the cohort of patients put on treatment one year earlier is made by the nurse under the responsibility of the medical doctor. A visit is then made by the supervision team; and the report is checked (and corrected if necessary) before being brought back to the coordinator for national statistics. To date, the notification system is paper based, but the programme is about to move to an electronic notification system (District Health Information System 2 developed by the University of Oslow).

*TB/HIV Coinfection Management*. Since 2006, all TB cases in the country have been systematically offered HIV testing. Those who are found coinfected with TB and HIV receive cotrimoxazole (CTX) for opportunistic infections prevention. Since 2010, antiretroviral therapy (ART) has been initiated for all coinfected TB/HIV patients, regardless of their CD4 cell counts [11].

*Financing*. TB activities are funded by the government. However, similar to many other developing countries, an important component of the funding is provided by the International Institutions, especially the Global Fund against AIDS, Tuberculosis and Malaria (GFATM). In 2014, 48% of the national programme needs were internationally funded [12].

### 2.3. Study Population

All TB patients registered in the NTP between 2000 and 2014 were included in the study.

### 2.4. Data Variables, Data Sources, Data Validation, and Definitions

Aggregate data were collected for this study and included number of presumptive TB cases, number of notified TB cases (all types), number of NPBCT cases (patients never treated or treated with anti-TB drugs for less than 1 month, whose TB diagnosis was bacteriologically confirmed on sputa by smear microscopy, culture, or WHO-approved rapid diagnostics such as Xpert MTB/RIF), new pulmonary clinically diagnosed TB (patients with pulmonary disease never treated or treated with anti-TB drugs for less than 1 month who did not fulfil the criteria for bacteriological confirmation but were diagnosed with active TB by a clinician who decided to give the patient a full course of TB treatment), new extrapulmonary TB (patients never treated or treated with anti-TB drugs for less than 1 month with a bacteriological or a clinical diagnosis of TB involving anatomical sites other than the lungs), retreatment TB (or previously treated patients who received 1 month or more of TB treatment in the past. They included relapse, treatment after failure, and treatment after loss to follow-up cases) [13], demographical characteristics of NPBCT cases (sex, age group, and department), and HIV-status of NPBCT (positive, negative, and unknown). These data were collected into paper based quarterly reports and validated during quarterly supervisions. They were then entered into a Microsoft Excel file.

### 2.5. Analysis and Statistics

Baseline characteristics, types of TB, and HIV-status were described using frequencies and percentages. The notified NPBCT rates in males, females, age groups, and departments were calculated from the general population. For the time series analysis, the number of TB cases notified half-yearly between 2000 and 2014 was derived. The “R” software version 3.2.1 (Institute for Statistics and Mathematics, Vienna, Austria) and the Box-Jenkins (1976) modeling approach were used to analyse the time series and to determine the best suitable model for the time series data. The Ljung-Box-Pierce test was performed to check whether the model was correctly specified. Levels of significance were set at 5%.

### 2.6. Ethical Considerations

Permission for the study was obtained from the NTP management staff. Approval from the local Ethics Committee “Comité National d'Ethique pour la Recherche en Santé” (http://www.ethique-sante.org/index.htm) was not required because of the retrospective nature of this study according to the country's recommendation. Approval was also not required from the Ethics Advisory Board of the International Union against Tuberculosis and Lung Disease (The Union) because of the use of aggregate not individual data. The study used already collected data. Therefore, written informed consent given by participants was not possible to obtain. Since aggregate data were used for the study, there was no way to recognize participants.

## 3. Results

### 3.1. Cases Detection and Types of TB

**Figure 1:**
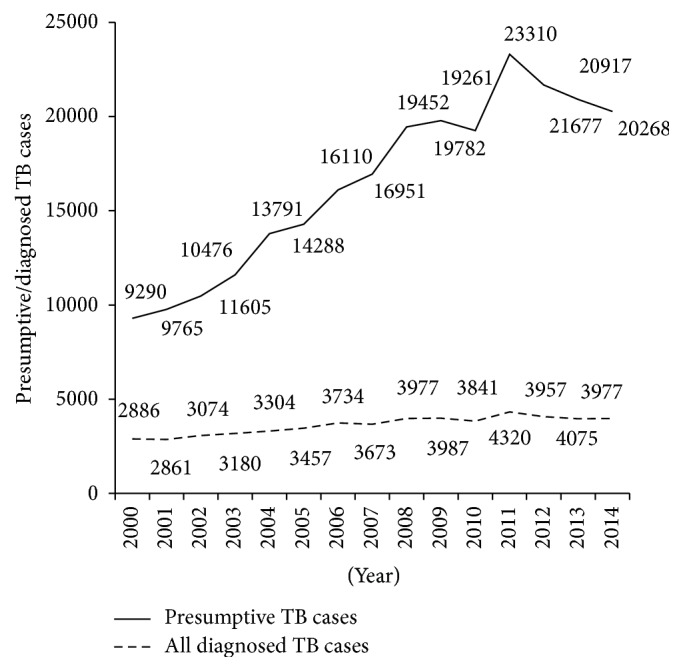
Trend of presumptive and notified tuberculosis cases in Benin, 2000–2014.

**Figure 2:**
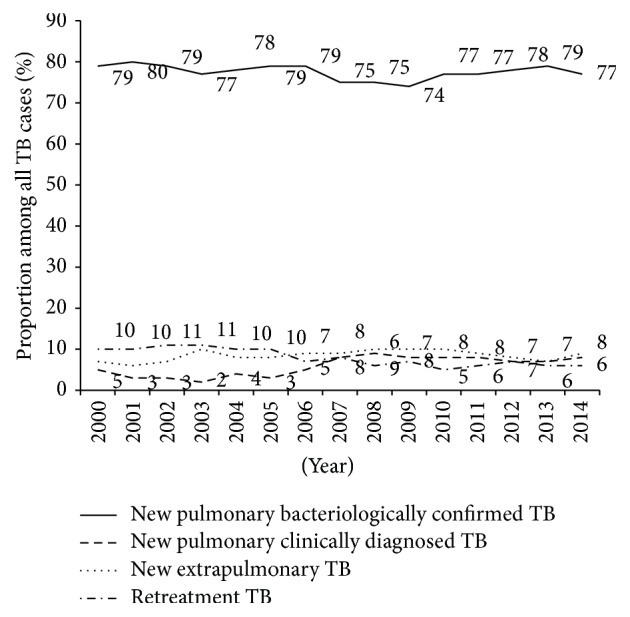
Different types of tuberculosis diagnosed in Benin, 2000–2014.

Between 2000 and 2014, of the 246943 TB presumptive patients, 54303 (22%) were diagnosed with TB. The numbers/trends of presumptive and diagnosed TB patients seen, respectively, over these fifteen years are shown in Figure 1. There was a progressive increase in the number of patients being investigated for TB. In the same manner, the number of diagnosed TB cases also increased. The maximum number of presumptive and diagnosed TB cases was reported in 2011 and this was followed by a decrease the year after.  Figure 2 shows the different types of TB diagnosed over the study period. NPBCT through microscopy represented the large majority of TB cases diagnosed (average = 78% [±SD 2%]). Retreatment TB patients declined from at least 10% in the first five years to about 6% in the last few years while new pulmonary clinically diagnosed TB cases slightly increased from 2% to 8%.

### 3.2. Baseline Characteristics and HIV-Status Variation

**Figure 3:**
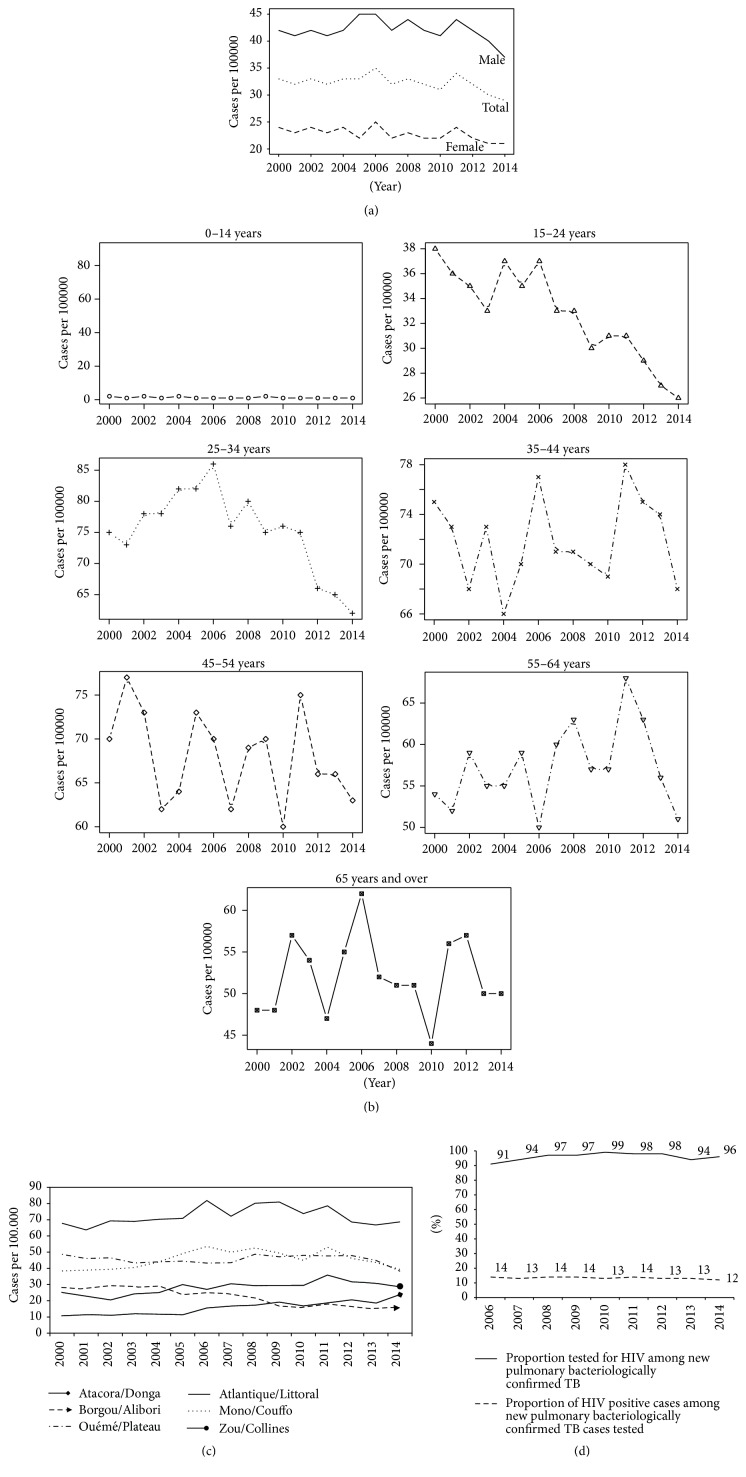
Trend of baseline characteristics and HIV positive status of tuberculosis patients between 2000 and 2014, Benin. (a) Sex in NPBCT; (b) age group in NPBCT; (c) regions of diagnosis for all TB; (d) HIV prevalence in NPBCT.

Baseline characteristics and HIV-status of patients are shown in Figures 3(a), 3(b), 3(c), and 3(d). Among NPBCT, the notification rate was 1.5 times higher in males compared to females. However, from 2012 onwards there was a decrease in male case numbers (Figure 3(a)). TB case notification rates decreased among young people aged from 15 to 34 years compared with older people (Figure 3(b)). The Atlantique-Littoral region continuously reported the highest rate of TB during the last ten years while a decrease in cases was noticed in Borgou-Alibori (Figure 3(c)). The proportion of TB patients tested for HIV was high (>90%) while the prevalence of HIV positive status among NPBCT cases tested was quite constant each year at 13% (Figure 3(d)).

### 3.3. Seasonality of TB Case Detection and Forecasted Numbers over the Next Five Years

**Figure 4:**
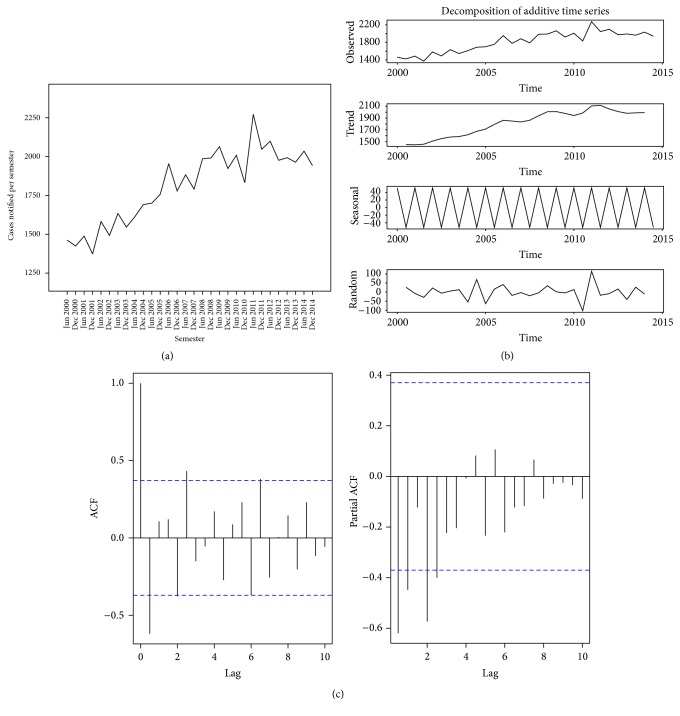
Time series analysis of all tuberculosis cases diagnosed, 2000–2014, Benin. (a) Tuberculosis cases diagnosed per semester; (b) decomposition of tuberculosis cases diagnosed with an additive model; (c) Autocorrelation and Partial Autocorrelation Functions of tuberculosis time series.

The time series analysis of TB cases diagnosed between 2000 and 2014 in Benin is shown in Figures 4(a), 4(b), and 4(c). The raw graph of all cases diagnosed each semester showed an overall gradual increase of these cases over the study period, with several peaks suggesting a cyclical seasonal pattern in TB case finding (Figure 4(a)). This assumed that the TB case finding time series was composed of a trend (gradual increase over time), seasonal variations (several peaks at regular intervals), and a residual component. After a profiles method, the additive model was the most appropriate to decompose this time series. The decomposition into trend, seasonality and residual components is shown in Figure 4(b). Trend analysis showed upward-sloping but not linear curve. The seasonality curve described an alternative and regular fluctuation in the interval (52.009; −52.009) over the study period. Overall, in each year, except 2004, 2005, and 2008, the number of TB cases notified for the first semester was higher than that reported for the second semester. The residual component was on average generally constant over the period. The Dickey-Fuller test confirmed that this time series was effectively stationary (Dickey-Fuller = −5.781, Lag order = 3, and *P* value = 0.01). The Autocorrelation Function (ACF) and the Partial Autocorrelation Function (PACF) suggested an Autoregressive Integrated Moving Average (ARIMA) (2,0, 3) model (Figure 4(c)). The coefficients of the autoregressive and moving average parts of the best fit model were determined and are presented in Table 1. The best fit model for this time series was therefore ARIMA (0,0, 1)(1,0, 0) with a constant. This model was validated using the Ljung-Box-Pierce test (chi-squared = 11.939, df = 1, *P* < 0.001), confirming thereby that the residual components in this time series were white noises (i.e., independent and identically distributed).

**Figure 5:**
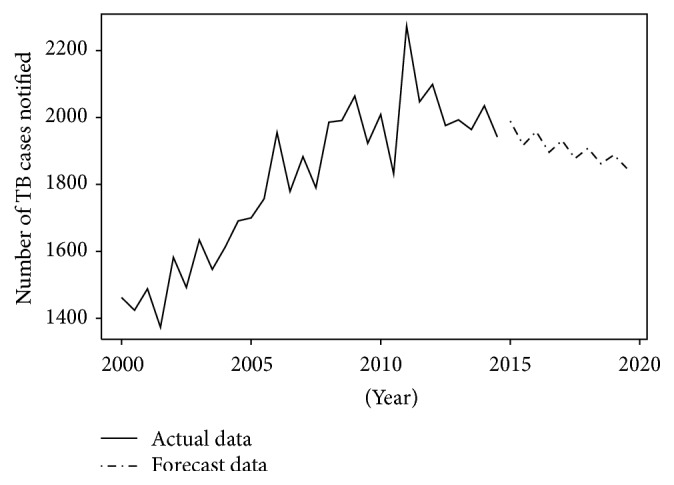
Trend of TB cases in Benin between 2000 and 2014 and forecasted number of TB cases from 2015 to 2019.

Finally, the forecasted number of TB cases per semester between 2015 and 2019 was calculated and is presented in Table 2. The numbers of TB cases are predicted to decrease during these next five years (see Figure 5).

## 4. Discussion

This study aimed to describe in Benin, at the start of the new Global End TB Strategy, changes that occurred in the epidemiology of TB patients during the previous fifteen years and the predicted trend in TB case finding in the following five years. We found that between 2000 and 2014, the number of presumptive and notified TB cases slightly increased. In contrast to new pulmonary clinically diagnosed TB cases that increased during this time period, the proportion of retreatment TB cases decreased. Patients who were found NPBCT positive on microscopy regularly comprised at least three-quarters of all notified TB cases. With respect to demographic characteristics, the burden of the disease was consistently higher among males compared to females. However, from 2012 onwards, there was a striking decrease in males being notified with TB. The burden of disease also decreased among younger adults aged less than 34 years, who also constituted a large proportion of the TB population. The Atlantique-Littoral region continued to drive the epidemic in the country, while there was a drop in notified cases in Borgou-Alibori.

HIV testing in TB patients was excellent, with each year over 90% of patients tested. Of those who were tested, one in seven was annually found to be coinfected, with no change observed in this proportion over these fifteen years. The findings showed a seasonal variation in TB case finding and notification, with a higher number of TB cases reported at the end of the first semester of each year. Finally, the forecasted number of TB cases to be diagnosed and notified in the next five years was predicted to decrease if only the current passive screening strategy in the country is applied.

The strengths of this study were that it involved all TB patients in the country who were notified to the NTP and therefore there was no need for any sampling framework. Data used were also previously validated during regular quarterly supervisions which contributed to minimizing errors. The study followed the Strengthening the Reporting of Observational Studies in Epidemiology (STROBE) guidelines [14]. Limitations were related to the retrospective nature of the study.

One of the major concerns that came from our findings was the decrease of TB cases in men and young adults and also the decline in the forecasted numbers that will be notified in the next five years. Taking into account the most important achievement within the programme, that is, a treatment success rate of 90% or more among NPBCT in these last six years [12], it is possible that the programme has significantly cut down transmission of infection from index smear-positive cases leading to a reduction in the burden of TB in the country. However, this belief should be interpreted with caution for several reasons.

First, the programme routinely uses a passive strategy for TB screening. The decrease reported in the study is therefore related to TB cases that reached health facilities. Unfortunately, the utilization of the national health services remains low, estimated at 51.4% in 2013 [15]. In other words, TB cases that occurred in those not accessing TB services will not be diagnosed and notified. The increase in notified TB cases reported in the year of the “TB Reach” experience followed by a decrease after this adds weight to this theory. Advantages of a semiactive case finding strategy have also been reported elsewhere [16, 17]. The high cost-efficacy of a semiactive case finding strategy does not allow it to be routinely performed in a resource-constrained setting, although this should always be considered. In 2015 and with the positive impact of the “TB Reach” experience on TB case finding, the GFATM authorized the implementation of other active case finding strategies in the country. An additional measure to be taken into account for reaching those who do not use health facilities could be to strengthen sensitization especially among traditional practitioners who, most of time, are at the front line for providing care.

Second, it is possible that patients of some vulnerable groups such as HIV-infected patients, those with diabetes mellitus, and pregnant women were not diagnosed, since they were not systematically screened by practitioners for TB at each visit, unless they complained themselves of presumptive TB symptoms. Actions to address this shortfall and also implementation of some semiactive case finding sessions have been planned in the next three years' national strategic plan, mainly with the new GFATM grant. All these new activities are expected to increase TB case finding as follows: 5093 cases by 2016, 5449 by 2017, 5613 by 2018, and 5781 by 2019.

Third, because of the strong relationship between TB and poverty, TB reduction/elimination undoubtedly will not be achieved without real improvements in population life conditions. Although the GDP per capital in Benin grew from US$ 339 in 2000 to US$ 810 in 2014, the country remains one of the poorest in the world, with a poverty headcount ratio of 36% in the general population [18, 19]. A significant improvement in population life conditions is probably needed to effectively reverse the TB trend in the country and to achieve the 2035 TB goal of ending TB epidemic.

The high predominance of NPBCT positive cases on microscopy with no apparent decrease over the 15 years confirms the laboratory network efficacy but also raises the question about early diagnosis of TB in the country. A positive result on smear microscopy requires an average concentration of 10000 bacilli per millilitre in sputum specimens [20]. There is a need to advocate for earlier screening and diagnosis through more education, communication, and effective screening of close contacts of smear-positive TB patients [17]. The progressive reduction in the proportion of retreatment TB patients is, however, a favourable observation and is likely to be due to improvements in treatment outcomes of new cases.

We are not surprised that the Atlantique-Littoral region which houses the economic capital and is burdened with rural migrations and promiscuity issues remains the one most affected by the disease; but reasons why the trend of TB notifications is declining in Borgou should be sought and addressed. In the same way, reasons for the apparent seasonality in notified TB cases in the country are not clear. One suggested hypothesis is the variation in health facilities utilization in relation to seasons. Access to health facilities in the rainy season is a problem in many countries, and it might be expected that a decrease in TB case notifications occurs at this time. However, this hypothesis is not supported by our data, since in contrast a higher number of notified TB cases was often reported during the first semester which is when the main rainy season occurs. There may be other determinants contributing to TB case finding and notification seasonality in the country. Seasonality in TB cases finding has also been reported from elsewhere [21–23]. One hypothesis found in the literature which attempted to explain such seasonality has been a variation in Vitamin D, important substance in host defence, which is produced by the body in association with sunshine exposure; it may be a factor [24].

With respect to HIV infection, the proportion of patients with HIV infection has not increased. HIV is an important driver of the TB epidemic in many countries. Benin is a mixed epidemic country. Between 2000 and 2013, there has been a decrease of HIV prevalence in the population aged between 15 and 49 years from 2% to 1.1%. Furthermore, the coverage rate of ART intake that started in 2002 has progressively improved. The proportion of HIV-infected patients on ART increased from 12080 in 2008 to 28850 in 2014 [6, 25]. All of these factors have probably helped in preventing an increase in HIV-associated TB in Benin during the last 15 years.

## 5. Conclusion

The number of TB cases over the last 15 years in Benin has decreased among males and young adults, and the number of forecasted TB cases predicted to be diagnosed in the next five years will also decrease if only a passive screening strategy is continued. Benin needs to decide whether it needs alternative case finding strategies to meet the End TB Targets by 2035.

## Figures and Tables

**Table 1:** Estimation of the coefficient of the best fit model for the TB case finding time series.

	MA1	AR1	Intercept
	0.6037	0.865	1759.7924
Standard error	0.2232	0.092	163.3689
Best fit model	ARIMA (0, 0, 1) (1, 0, 0) with constant		
Convergence criteria	Log likelihood = −182.67; AIC = 373.33; BIC = 378.94		

Note: MA: moving average; AR: autoregressive.

**Table 2:** Forecasted number of tuberculosis patients predicted to be diagnosed between 2015 and 2018 in Benin.

	2015		2016		2017		2018		2019	
	S1	S2	S1	S2	S1	S2	S1	S2	S1	S2
Prevision	1989	1917	1958	1896	1931	1877	1908	1861	1888	1848
[IC 95%]	[1788–2189]	[1683–2152]	[1666–2250]	[1586–2206]	[1587–2275]	[1522–2234]	[1529–2287]	[1475–2249]	[1485–2291]	[1439–2257]

Note: S: semester; IC 95%: 95% forecasting interval.
